# Understanding the Context and Needs of Adolescents Experiencing Subclinical Anxiety and Depression Symptoms in Wales: Document Analysis and Qualitative Data Collection

**DOI:** 10.1007/s12310-026-09849-3

**Published:** 2026-02-21

**Authors:** Hayley Reed, Rohen Renold, Erin Hewald, Yulia Shenderovich

**Affiliations:** 1 The Centre for Development, Evaluation, Complexity and Implementation in Public Health Improvement (DECIPHer), School of Social Sciences, Cardiff University, Maindy Road, Cardiff, CF24 4HQ UK; 2 The Wolfson Centre for Young People’s Mental Health, Cardiff University, Maindy Road, Cardiff, CF24 4HQ UK

**Keywords:** Indicated prevention, Mental health, Adolescence, Intervention

## Abstract

This study aimed to understand the context and needs of adolescents experiencing elevated but subclinical anxiety and depression symptoms, who have been termed the “missing middle” in Wales (UK). It is part of a project to identify and adapt an effective secondary school-based intervention to Wales. The methods used were document analysis, and qualitative primary data collection. Sixteen documents were analysed: Welsh policy (n = 2), primary research (n = 3), research overviews (n = 2), and reports of stakeholders’ views on adolescent mental health (n = 9). Next, focus groups and interviews were conducted with 35 students aged 11–18 years old; 18 school staff; 23 practitioners involved in managing, delivering or referring to non-clinical and clinical mental health services; and two policy officials. A hybrid approach of deductive and inductive thematic analysis was conducted which produced four themes. Firstly, we identified definitions and understandings of the “missing middle”. Secondly, contextual factors included an increase in help seeking, alongside challenges of self-diagnosis and the striving for specialist support even when this was inappropriate. A third theme highlighted enablers and barriers to implementing targeted provision e.g., while funding had increased for school services, this was still not adequate to meet demand. Fourthly, desired intervention targets were at the intrapersonal (cultivating emotional regulation and problem-solving skills) and the interpersonal levels (reducing loneliness and supporting social relationships), with school-based, one-to-one delivery via external providers preferred. These findings offer insights into how preventive interventions should include those experiencing high levels of distress and/or functional impairment even in the absence of high symptoms.

**Supplementary Information:**

The online version contains supplementary material available at 10.1007/s12310-026-09849-3.

## Background

### Indicated Mental Health Interventions

Adolescent mental health is a prevention priority. It is estimated that 13% of 10–19 year-olds have a diagnosable mental health disorder (United Nations Children’s Fund, 2021). Mental ill health is the leading cause of disability-adjusted life years in under 24-year-olds, with anxiety and depression being two of the most prevalent disorders (Erskine et al., 2015). The peak age of onset for anxiety disorders is 5.5 years old with a second peak at 17 years old, and for depression it is 20.5 years old (Solmi et al., 2022). These conditions can have chronic and relapsing trajectories (Moriarty et al., 2020), with relapse in 1/3 of those with anxiety (Taylor et al., 2015) and up to 3/4 of those with depression (Conradi et al., 2017).

Studies also report a growing global population of adolescents with subclinical but elevated anxiety and depression symptoms. As those with subthreshold symptoms are at higher risk for later disorder onset than those without symptoms (Fergusson et al., 2005), this has led to the growth in the development and evaluation of indicated interventions. Even prior to COVID-19, an increasing trend in adolescent emotional problems was demonstrated (Anthony et al., 2023; Collishaw, 2015; Patalay & Gage, 2019), but this has been exacerbated by the pandemic (Creswell et al., 2021; Racine et al., 2021; Viner et al., 2022). One systematic review from 2022 found 17 studies that measured anxiety symptoms and 25 that measured depressive symptoms that showed population increases from pre to post pandemic (Samji et al., 2022).

Reports by the US Institute of Medicine (Mrazek & Haggerty, 1994; O'Connell et al., 2009) demarcated primary prevention interventions as those provided before the first onset of a diagnostic disorder, whereas treatment interventions aim to cure the disorder or reduce symptoms in those with a diagnosed condition. Primary prevention interventions include universal, which are delivered to a whole cohort, selective, which target groups or individuals with an elevated risk, e.g., parental psychopathology, and indicated, which target populations with early symptoms or behaviours that precede disorder. Selective and indicated interventions are collectively referred to as targeted interventions (e.g., Hetrick et al., 2016; Werner-Seidler et al., 2021).

Indicated interventions fall between universal programmes, that tend to focus on psychosocial content such as social emotional learning for all adolescents, and treatment programmes, that focus on therapeutic approaches such as cognitive behavioural therapy delivered mainly in clinical settings. Indicated interventions are often delivered in school settings due to the potential reach in the population, and are shown to be more acceptable to deliverers and recipients as they can often be tailored to the individual needs of adolescents, and they can be more sustainable (Fazel et al., 2014; Rapee et al., 2006).

There is evidence that indicated interventions are effective in decreasing secondary school students’ anxiety and depression symptoms (Werner-Seidler et al., 2021; WHO & UNICEF, 2021). Further, targeted approaches (indicated and selective) tend to have higher effects sizes than universal programmes (Werner-Seidler et al., 2021). Providing universal interventions alone may not be sufficient to alter the trajectories of those already exhibiting symptoms (Greenberg & Abenavoli, 2017), with one study finding a possibility of increased emotional difficulties for some as they become aware of their symptoms (Montero-Marin et al., 2022). Researchers have highlighted the need to explore for school-based interventions “what works, for whom and how, as well as considering key contextual and implementation factors” (Kuyken et al., 2022, p.104).

### Incorporating Lived Experience in Indicated Prevention

This study is important as it adds to the emergent literature on including the voices of those with lived experience in indicated prevention. Qualitative research on depression demonstrates that adolescents’ lived experience of mental health difficulties may not always match the expectations of professionals and map onto diagnostic criteria (Twivy et al., 2023) which demonstrates the need to incorporate lived experience. However, within the indicated prevention field there is a lack of qualitative research with or about the adolescent population with subclinical depression and anxiety symptoms. For example, Beket et al’s (2025) review of qualitative studies on adolescents’ perceptions on mental health found 74 articles but only one aimed explicitly at understanding subclinical depression, while a few others focused on experiences of emotional distress, and seeking/accessing help, including barriers to support, that may be associated with this group.

Further, indicated interventions have historically been developed by researchers or adapted from treatment approaches without incorporating lived experience. There is nascent literature about the use of university-community partnerships that utilise human-centred design (HCD) to develop and adapt school-based mental health interventions (Lawson & Owens, 2024). The first step in HCD processes often employs predominately qualitative methods to elucidate the lived experience of stakeholders, such as students and school staff, as a means of “discovery of user and context needs” (Lyon, 2024, p. 932).

Therefore, both the presentation of subthreshold mental health difficulties and the indicated approaches to addressing them, require the understanding and integration of lived experience. Our work does this through primary qualitative data collection that centres the perspectives of stakeholders including adolescents and school staff, and analysis of documents comprising those that foreground stakeholders’ views on adolescent mental health in Wales. This will provide insight into the support needs of young people experiencing subclinical levels of depression and anxiety within the context of Wales for the purpose of adapting an intervention for the “missing middle”.

### Adolescent Mental Health in Wales

Wales is one of the four UK nations with devolved responsibility (transfer of decision-making power and resources from the central government to a country in a union) for health. The growing burden of adolescent anxiety and depression shown globally is reflected in Wales. This is demonstrated through biennial repeated cross-sectional, representative samples of Welsh 11–16-year-olds that undertake the Strengths and Difficulties Questionnaire which includes questions on mood and fears. In 2019–20, 39% of adolescents reported at least slightly elevated mental health symptoms (Page et al., 2021), and this increased to 46% in the 2021–2022 survey (Page et al., 2023). Pre-pandemic analyses also show these increases in emotional problems are observed throughout the secondary school age group (11–16 year olds), with more marked increases for girls and those from families with higher deprivation (Anthony et al., 2023).

There has been a call to reform the Welsh mental health system to focus on the early identification of adolescents’ emotional and mental health before problems escalate, especially as there is concern that the “missing middle” often do not receive appropriate support (Children, Young People and Education Committee, 2018). The “missing middle” has been used to refer to the population of adolescents in Wales not reaching the service threshold for specialist care; however, a clear definition of this population is elusive. At study inception, the “missing middle” were considered synonymous with adolescents who present with elevated anxiety and depression symptoms, but this will be explored further in this paper. Since the term “missing middle” appears to be introduced in policy documents without drawing on consultations for this concept, this study tests and explores the concept with young people, school staff and wider stakeholders in the education and health sectors.

In 2021, the Welsh Government made it statutory for schools to embed a Whole-School Approach to Emotional and Mental Wellbeing (WSA) which, amongst other activities, requires schools to offer targeted programmes. This policy does not distinguish between indicated and selective intervention approaches but simply advocates for offering targeted approaches to those that “need support over and above universal provision” (Welsh Government, 2021, p. 45). Schools are tasked with providing a range of targeted provision dependent on need and within school settings, where possible and appropriate.

Meeting this statutory requirement for targeted programmes is difficult for schools in Wales. Whilst there is an effective international evidence base, there is relatively scarce evidence from the UK (Cheney et al., 2014). One international review (Clarke et al., 2021) found only five UK based anxiety and/or depression prevention studies and they showed no effects (Barry et al., 2017; Weeks et al., 2017), small effects in methodologically poor studies (Pearce et al., 2017; Putwain & Pescod, 2018) or studies with relatively short follow-ups (Brown et al., 2019). There is also a lack of understanding nationally about the targeted provision schools offer, a gap our study contributes to addressing.

### Study Overview

The study is part of a wider project that will select a school-based indicated intervention to adapt to Wales following the ADAPT guidance (Moore et al., 2021). This and other intervention guidance (Craig et al., 2018; Skivington et al., 2021), and the growing literature on best practice for the development and adaptation of school mental health interventions/services through university-community partnerships (Lawson & Owens, 2024), highlight the importance of understanding intervention needs and theory, and the context interventions will be delivered in to support future implementation.

This study aligns with the university-community partnership movement in the following two ways. Firstly, it draws on research methods to understand the lived experience of a range of stakeholders in Wales, and includes the views of a range of advisory partners on the conduct of the research through a Study Advisory Group (SAG) and two Young People’s Advisory Groups (YPAG). The SAG has 10 members from academia, policy and practice. The two YPAG’s are pre-established groups set up by research centres at Cardiff University. One from the DECIPHer centre (called ALPHA) who are a group of 15 young people aged 14–25 years old; some of which have tried to access targeted support in school. The other is part of the Wolfson Centre for Young People’s Mental Health and includes 12 young people aged 14–25 who have lived experience of mental health conditions and services. Where these groups were involved is noted throughout this paper.

Secondly, it is driven by implementation science principles as it has a focus on understanding the context an intervention will need to fit into as well as the intervention characteristics. This is particularly relevant for the implementation of mental health interventions within schools as this can be complex and challenging (Gee et al., 2021). The Context and Implementation of Complex Interventions (CICI) framework’s (Pfadenhauer et al., 2017) context and intervention dimensions were used to conceptualise these. This draws out the seven context domains of epidemiological, ethical, geographical, legal, political, socio-cultural, and socio-economic, and the three intervention domains of theory (how the intervention makes change, e.g., through psychoeducation), delivery (how and where, e.g., the delivery agents) and the design (what is delivered, e.g., group-based vs individual). This was used with a socio-ecological model (McLeroy et al., 1988) to frame the levels interventions can target which include the intrapersonal (an individual’s characteristics such as their knowledge), interpersonal (an individual’s social relationships), organisational (social institutions such as schools), community (broader context including social norms and the physical environment), and policy (the local, regional and national policies and laws) levels.

Therefore, this foundational study focuses on understanding the Welsh adolescent mental health context, and the needs of the “missing middle” population of secondary school students aged 11–18. The research questions we address are:

1. Who are the “missing middle”, as defined by documents, and perceived by young people and practitioners in Wales?
2. What is the current context in terms of targeted intervention for adolescent mental health in Wales?
3. How can the “missing middle’s” needs be addressed by a school-based intervention?

## Method

The study utilised two methods. First, document analysis was conducted on policy, research, and reports of adolescent mental health in Wales. Following this, focus groups and interviews were conducted with key stakeholders, including secondary school students, school staff, and staff in the health and educational policy and practice workforces, between March and August 2023.

### Document Analysis

Sixteen documents (see appendix 1, Table 2, for included documents) were located through searches conducted on relevant websites and citation tracking by two members of the team independently. Meetings were held to discuss and decide on documents found based on the inclusion criteria. Documents were eligible if the population they discussed were adolescents (young people, secondary school students), and the focus was on mental health and wellbeing, including key words about the “missing middle”. We included documents focusing on Wales and published in English since 2018, when the “missing middle” were first recognised in Welsh documentation. A consensus decision by two team members to stop searches was taken when no new content was located. Documents were classified by the study team as policy documents (n = 2), primary research (n = 3), research overviews (n = 2), and reports based on stakeholder views of adolescent mental health (n = 9).

### Interviews and Focus Groups with Secondary School Students and Staff

Schools were recruited through e-mails sent via the School Health Research Network in Wales (Murphy et al., 2021). The profiles of the three recruited schools are shown in Table 1. The schools were selected to vary based on size, percentage of students with free school meals entitlement, language of teaching and geographical location in Wales. Location data has not been included to preserve anonymity. Each school signed a School Research Agreement, outlining the responsibilities of the research team and the schools, including the safeguarding and ethical arrangements, and asking schools to nominate a study link teacher.

**Table 1:** Profiles of Schools

	Population size	Free school meal entitlement	Language
School A	1079 pupils (+)	21% (+)	English
School B	1879 pupils (+)	11% (−)	English
School C	601 pupils (−)	9% (−)	Bilingual

+  = above Welsh average; − = below Welsh average

Six focus groups were conducted with 35 (14 male and 21 female) secondary school students, with an average focus group size of 6 (range 3–10) students. The two focus groups conducted in each school were split by age (11–15-year-olds; 16–18-year-olds) and lasted between 32 and 49 min. Within schools, students were invited to nominate themselves.

Discussion with 11 young people (aged 15–21; female = 5, male = 3, other = 3) from the DECIPHer YPAG took place before the focus groups and influenced the research design. First, young people advised the team not to ask students about their understanding of the term the “missing middle” as it would be meaningless to them. Second, the YPAG developed the following definition we could use to explain the “missing middle” in study documents and focus groups—young people who do not have a mental health illness but who may be starting to feel a bit unhappy or anxious. Third, young people thought that the focus groups should be participatory/activity based. So a written vignette was developed using recent best practice evidence (Erfanian et al., 2020), and following previous studies on adolescent mental health with under 18 year olds (Kendal et al., 2011).

The full focus group schedule for students is available in appendix 2. The vignette of a young person dealing with early anxiety symptoms was read to students to open discussions and normalise the experience, so students talked in a more generalised manner about the target population rather than disclose their own mental health status. Following this, a socio-ecological model (McLeroy et al., 1988) was used to generate discussion about the help/services on offer, the quality of these, and if there were any gaps for the “missing middle”. Lastly, students discussed the acceptability and relevance of intervention elements the research team had prepared. This included discussing the potential intervention targets (the use of psychoeducation and problem-solving), delivery agents (teachers or those external to the school), and delivery design (group vs individual).

Eleven individual interviews and two focus groups (M = 2.5, range 2–5) were conducted with 18 school staff from the three schools. Invitations were sent to staff via school email systems from the school link teacher. Those who took part included three senior leaders, four heads of year, two curriculum leads, five pastoral staff, three learning support workers and a school nurse. Semi-structured interviews and focus groups followed guides asking open ended questions about their understanding of the current context in Wales in terms of targeted support, what the term “missing middle” meant to them, who was a part of this group, their mental health needs and how these needs could be supported. The interviews and focus groups were concluded by asking questions about the acceptability and relevance of proposed intervention elements (as with students). Research encounters lasted 22–57 min.

### Interviews and Focus Groups with Key Policy and Practice Stakeholders

A combination of purposive and snowball sampling was used. Purposive sampling identified stakeholders and organisations through the document analysis, and existing contacts of the SAG and the Research Centre staff where the study was hosted. Snowball sampling with participants was used to identify further organisations or individual’s central to adolescent mental health in Wales. A total of 79 individuals or organisations were contacted to take part. Participants were education and health policy officials (n = 2), and practice stakeholders (n = 23) involved in the management, development, delivery, or referral of adolescents to non-clinical and clinical services. Eleven individual interviews and three focus groups (M = 4.6, range 2–9) were held face-to-face at participants’ workplaces or via an online platform, dependent on participant preference. Topics covered and procedures were the same as with school staff. Research encounters lasted between 42 and 58 min.

### Ethics

Ethical approval for the study was provided by Cardiff University School of Social Sciences Research Ethics Committee. To ensure study materials and practices were acceptable and clear, all student documentation as well as recruitment and focus group strategies were reviewed by the DECIPHer YPAG (in the same group session discussed in the section about interviews and focus groups). Participants were provided with written bilingual (English/Welsh) information sheets and consent forms. Bilingual parent/guardian information sheets and consent forms were completed by a legal parent/guardian for each student involved. For student participants, study information was also verbally reiterated before formally beginning focus groups, and the option to withdraw was given. For adult participants, study information was verbally reiterated, and consent was recorded before interviews or focus groups.

### Data Analysis

A hybrid code book approach of deductive and inductive thematic coding (Braun & Clarke, 2021; King & Brooks, 2018) of the eligible documents, and interview and focus group data was conducted. The deductive codes were from CICI framework’s context (ethical, epidemiological, geographical, legal, political, socio-cultural, and socio-economic) and intervention (theory, design and delivery) dimensions (Pfadenhauer et al., 2017). The context domain was used to code data related to research question 2 about the wider current contextual factors and how this affects implementing targeted provision. The intervention dimension was used to code data for research question 1 and 3 to inform understanding of the population and the potential intervention characteristics.

Document analysis took place before primary data collection. For the document analysis, 25% (n = 4) were coded by the lead researcher and a research assistant independently. As some documents included irrelevant information (e.g., in-patient care), a decision was made to code only data relevant to the study and target population. Inductive data subcodes were discussed for consensus. The lead researcher then coded the rest of the documents and created a memo summarising the data into the CICI framework domains. In the memo, ten subcodes were developed in the intervention dimension (six specifically about the “missing middle” population (research question 1) and four about the theory and delivery of the intervention to meet population needs (research question 3)), and 20 subcodes for the context domain (research question 2). These subcodes informed the interview topic guides, e.g., questions about help seeking were added as this code found high help seeking behaviours reported for specialist mental health care alongside a lack of help seeking as young people believed they would not receive targeted support.

All interviews and focus groups were transcribed by an external transcription company and quality checked by the research team following the study’s data analysis plan. The primary data collected was deconstructed into stakeholder types to compare different opinions. Subcodes were generated based on the CICI framework, the subcodes from the document analysis, and notes taken during data collection. Three researchers coded one transcript for discussion and refinement of codes. All other transcripts were coded by one researcher for each stakeholder group. The researcher continuously collapsed and refined subcodes and grouped them into themes that were verified by other researchers in the team. The lead researcher wrote the summaries of each subcode and the overarching themes for each stakeholder groups which were verified by another researcher. The lead researcher combined the primary data themes and subcodes to the document analysis memo which was plotted by research question (see above) to produce a final summary of all the data.

Findings were presented to the SAG and two YPAGs for comment and review, with no major changes made to the main themes post these discussions. This included five members of the SAG, eight members of the Wolfson YPAG (aged 15–20; female = 5; male = 3) with lived experience of mental health conditions and services, and 10 young people from the DECIPHer group (aged 14–22: female = 6; male = 2; other = 2).

## Results

This section presents the combined key findings from the document analysis and stakeholder interviews. It first considers the understanding of the “missing middle” in Wales to address research question 1. For research question 2, it outlines the contextual factors surrounding this population and the barriers and enablers to providing targeted provision. It finishes by presenting the needs of the “missing middle”, and hence the elements that targeted programmes could focus on (research question 3).

### Understanding the “Missing Middle”

This section addresses research question 1 by providing an understanding of the “missing middle” through discussing the definition of the term and how the “missing middle” may present, the recognition of the increase in this population, and the wider social determinants that are perceived to influence this population’s mental health.

#### Definition

The “missing middle” was first discussed in a 2018 Welsh Government Scrutiny report by the Children, Young People and Education Committee:

*The vast majority of witnesses commented that urgent work was needed to address the lack (and in some cases absence) of services for children and young people who need support but do not meet the threshold for the specialist Child and Adolescent Mental Health Service or Neurodiversity support (p. 76).*

Throughout the document review, focus groups and interviews, the “missing middle” were defined by two factors. First, as shown above, there was a clear systemic issue as this group faced difficulties accessing appropriate support in Wales. Individuals were often*“bounced between services who cannot agree who is responsible for their care” (*Children’s Commissioner for Wales, 2020, p. 7) which may exacerbate adolescent needs if they internalise the rejection, and feel like the problem that cannot be fixed. This highlights the existence of multiple siloed support systems in education, health and social care. Stakeholders outside education mostly recognised but disliked the term as their understandings aligned with this being a systematic issue where the service(s) rather than the young people were missing. School staff were less likely to know the term.

Secondly, they are young people characterised as ‘mild to moderate cases’ in terms of mental ill health, however there were some mixed views on what this meant. Most participants recognised the “missing middle” as adolescents with mood disorder symptoms that did not reach diagnostic thresholds, but they could also be in high levels of distress and/or have impacted everyday functioning with lower levels of symptoms. For example, adults described some students that were unable to develop or maintain positive relationships with peers or school staff, but they did not attribute this to students worrying excessively.

Some sources included neurodiverse (ND) young people (those on the autistic spectrum or with attention deficit hyperactivity disorder) who struggled to receive adequate ND support in this population too, while others thought ND young people were more likely to suffer from anxiety symptoms which made them part of the “missing middle”. Others more likely to be part of the “missing middle” were those who are care-experienced, LGBTQ + (lesbian, gay, bisexual, transgender, queer or questioning, or another diverse gender identity), or those from more socioeconomically disadvantaged backgrounds.

#### Presentations

Related to how the “missing middle” was understood, it was found that they could present in several forms including young people who were introverted and isolated from peers, exhibiting behavioural problems, and young people who were infrequently attending school or who were complete school refusers. There was a subgroup of adolescents, which stakeholders’ thought were less visible:

*we've talked as a school about what we call the grey child, which is the ones that kind of come into school, they get on with it, they don't get too much attention, either negative or positive, and kind of go under the radar a little bit. And I would say that this type of pupil for mental health support could be overlapping. (School Staff)*

As these young people appeared to function well, they were less likely to be identified as in need of help. Young people thought these adolescents felt like they did not want to be a burden or that they were not suffering as much as their peers, so they did not seek help.

#### Recognising the Increase in the “Missing Middle”

There was consensus from all data sources about the increased numbers of adolescents within the “missing middle” population. Young people reported having multiple peers who were struggling with symptoms or to do everyday tasks, which often emerged at challenging times such as school transitions. School staff noted the need to monitor and check in on more students than in previous years, and the increase in the use of ‘privileges’ to allow students to leave class when they felt unable to cope. Practitioners discussed large increases in referrals to specialist and non-clinical services. Schools knew of growing numbers seeking help even though some documents highlighted help seeking was stemmed due to young people believing they would not receive support. School staff believed older students were presenting with more anxiety and depression related issues, whereas professionals felt presentations were beginning earlier. Students agreed that secondary schools focused on older student problems but needed to recognise and provide more support for younger students.

#### Wider Determinants

Related to how the “missing middle” is defined and understood (research question 1), views were shared about the perceptions of the wider determinants that influenced this population’s mental health. For example, while the increase in the “missing middle” population was evident pre-COVID-19, there was widespread agreement the pandemic had exacerbated issues and enlarged this group:

*there is little doubt that its [COVID’s] wider effects―and the measures taken to manage it―have impacted their lives significantly. These wider effects have been described to us as the “collateral damage” to children and young people caused by the pandemic, and include anxiety about periods away from school, clubs, family and friends (Welsh Parliament, 2020, p.10).*

Policy documents and participants argued that the pandemic had interrupted adolescents in an important developmental and educational period when they should have been learning how to cope with stress and establishing supportive relationships with peers and school staff. This was believed to have increased social isolation and screen time as well as affecting how safe adolescents felt in general. Difficulties were demonstrated through diminished school attendance rates still evident two years after national lockdowns had ceased.

The following three wider determinants were also discussed, with recognition that COVID had exacerbated these as well. Firstly, school transitions and examination pressure:

*“If you're going through exams and you do have stress, and you …. have like a lack of access to like support organisations or methods, I think if you can't learn to control that stress and anxiety from the exams, it could have like a domino effect”*. (Student)

Students also felt school staff and parents compelled them to achieve, and adolescents feared failure especially as some pupils thought they had not focused enough on their schoolwork during COVID. Secondly, family problems, which ranged from a lack of parenting skills to adverse childhood experiences, such as domestic violence. Thirdly, social media and technology as a source of pushing unrealistic standards for young people to meet, and to compare themselves to.

### Contextual Factors

This section partly addresses research question 2 about the wider current context for targeted intervention. The two themes discussed were: an increase in help seeking; and the challenges of self-diagnosis and the strive for specialist support.

#### Increased Help Seeking

Linked to the current context in Wales (research question 2), participants believed there were more opportunities for the adolescent population to discuss mental health, and school staff felt that most students notice when they are exhibiting mental health symptoms. A positive perception was that more young people who ‘genuinely’ have anxiety and depression symptoms are now help seeking in Wales, even if they find it hard to access an appropriate service. This was showcased above through school staff reporting more students seeking help in schools and practitioners noting increased referrals to specialist services. Overall, it was felt that there was less stigma between young people to discuss if they are struggling or if they have sought help, however it was noted that young people are living alongside an older generation that are not always as open and aware of mental health. Stigma was still present, particularly in rural and some ethnic communities:

*There's a lot of stigma, I find, especially in the older generation and in certain communities. Like, I know in the Asian community, mental health isn't prioritised. It's often seen as, you know, quite trivial and something you deal with on your own. (Student)*

It was discussed that young people from these communities may only seek help if their families were not informed or they would avoid seeking help so not to damage family pride.

#### Self-diagnosis and the Strive for Specialist Support

One negative raised about the increased awareness of mental health was the tendency for some young people and their families to label normal worries, sadness and developmentally appropriate mood fluctuations as having anxiety and depression.

*So rather than feeling that everything is anxiety, everything is depression, that we have these normal ebbs and flows in life and not everything is always going to be perfect and not everything can be fixed with using a pill or a drug. So more sort of normalising of being a human being, you know? (Policy and Practice Stakeholder)*

For some students and their families this lack of understanding of normal emotional fluctuations manifested itself as self-diagnosis. Practitioners and school staff spoke of parents who said their children had anxiety but when questioned it was clear no diagnosis had been undertaken by a clinician. School staff also spoke about social contagion which they described as groups of students within schools who had all self-diagnosed as having the same mental health issue and consistently report the same symptoms. A few participants thought self-diagnosis was increasing as growing up was being medicalised and gave examples of language that evidenced this, such as ‘social prescribing’. Documentation corroborated this: *“pupils’ resilience was declining and normal childhood experiences, such as sadness, were being medicalised”* (Holtom et al., 2021, p. 43).

Practitioners discussed unpicking referrals to de-medicalise information provided, and they thought self-diagnosis was shown through families striving for a referral to specialist services such as the CAMHS (Child and Adolescent Mental Health Service) as they thought this was their ‘golden ticket’ to solve the problem. Often practitioners thought referrals were inappropriate as the adolescent was experiencing subclinical symptoms or because the problem was located within the family.

### Implementing Targeted Provision

This section further addresses research question 2 about targeted intervention in Wales through exploring the enablers and barriers to implementing this provision from outside school contexts (policy and financial influences) and within them (processes for identifying and assessing need, and providing the services required).

#### Policy and Financial Influences

The WSA mandated schools offer targeted provision with a robust or emerging evidence-base. Alongside the WSA framework, policy documents such as the Delivery Plan for Mental Health and the Development of a Theory of Change for the WSA continue to advocate for targeted provision. However, a barrier to implementing the WSA raised by those outside of the education sector was how it linked into other important policy progressions such as the NEST (Nurturing, Empowering, Safe and Trusted) Framework. This is a cross-sector tool for the development of mental health services for all. It focuses on joint sector working but wider stakeholders thought separate frameworks for education and the whole system perpetuated siloed working. This was also demonstrated in the data as school staff did not mention NEST.

One enabler was the extra funding provided to facilitate school-based targeted provision, for example:

*£626,000 of additional funding was made available for the financial year 2019-20 to address high demand and long waiting lists. In April 2020, the Minster for Education committed an additional £1.25 million to counselling services for the 2020-21 financial year to deal with an anticipated increase in demand as a result of the COVID-19 pandemic. (*Hewitt et al., 2022*, p. 6)*

However, barriers raised by schools included budgets still not meeting demand, being unsure how to allocate budgets to mental health provision, and differing views on a school’s responsibility for mental health:

*So for every headteacher that will say, "Let's fund a school-based counsellor," there'll be a headteacher that says, "Well, no. It's our responsibility to manage the budget in this way. These are our priorities. And I'm sorry to say that the local authority or whoever else will have to plug the gap. (School Staff)*

#### Identifying and Assessing Need

As described above, participants reported an upsurge in the numbers of young people needing extra support. Students emphasised help seeking was further enabled where there was a supportive school environment which included having adults they can trust. School staff advised that they strived to create open, safe environments, with strong student-staff relationships, where students could reach out if needed, and they would be listened to and treated as individuals. Barriers to achieving this were the limited time and capacity within schools, and students noted that not all staff members were approachable. Some students thought the onus should be on schools to identify those who are struggling e.g., they wanted *“Someone who approaches you. 'Cause every time you need to talk to a teacher, you approach them”*. (Student).

School stakeholders discussed informal processes of identifying students in need, such as sending emails to pastoral staff or form tutors and formal processes through tracking systems to raise their concerns, and surveys:

*We do the PASS [Pupils Attitudes to Self and School] survey, which we kind of get a bit of a benchmark of where all the pupils are in the school. And then immediately, we're able to put in interventions for not necessarily the highest tariff pupils, who have usually all got some support going in for them, but the next layer down, children that we wouldn't necessarily have thought there were issues. (School Staff)*

Tracking systems were used to collate information from different staff members to piece together whether a student should be approached to offer them support.

A new structure in the education sector called CAMHS In-Reach had been developed to facilitate assessing adolescents’ support needs. These teams hold case consultations with school staff (but not always with young people directly) to advise how to support or signpost identified adolescents to services that fit their needs. Study participants from this workforce stressed gathering information to contextualise cases rather than relying on assessment or symptom checklists.

*And I do a screening or create a discussion. I can be there in two hours 'cause I have to know all about the family. It’s quite often people want to fix little Johnny. And I'm like, "No, I need to know everything else that's going on," you know. (Policy and Practice Stakeholder)*

Wider stakeholders stressed this structure could stem inappropriate referrals to specialist services, ensure referrals incorporated necessary information, and ensure adolescents accessed mid-tier support in a timely fashion. However, they also expressed variability in school uptake, and some school staff advised they did not know this workforce existed or did not engage with it. A few school staff reported high demand for CAMHS In-Reach and positive experiences with the support received, claiming this gave them insight into how to support individuals; whereas others thought the service remit and practitioner capacity needed to be expanded into providing the targeted service (which in some areas it had).

There was concern that, underpinned by the NEST Framework, new multidisciplinary panels had also been developed to assess adolescent support needs. These had advantages such as they strived to get the young people’s views on their needs, but educational representatives were not always on these panels as desired, and the panels could not signpost into school services. This has the potential to reinforce the systemic issues discussed earlier.

#### Current Service Offer

Through question 2, the research also sought to understand the current service offer for targeted prevention. It has already been highlighted that there was a concern that an appropriate service offer did not exist for the “missing middle” in Wales in 2018 (see section entitled “Definition”). However, in this study, there was a perception from students and adults that schools were offering an abundance of support in-house such as counselling, pastoral teams (with varying programmes of work), and emotional literacy support assistants trained in interventions. As was key in the WSA, this was enabled by schools engaging with a range of organisations offering non-clinical mental health support from the voluntary, health and social care sectors, and for-profit companies. One participant noted though:

*Schools are a very crowded marketplace in terms of lots of people wanting to rush into schools, offer support, offer this, offer the other, do this workshop, do this, "We can come and do this. We can come and deliver this. We can do this with your staff. We can do this with your children." Actually, that's not a whole school approach. That's a pellet gun that's sending lots of pellets to the same place. (Policy and Practice Stakeholder)*

Other issues were raised with the service offer as follows. Firstly, there is not always clarity on what each service/intervention offers, and no coordination of services so it is difficult for schools to understand what provision is appropriate for students. This may become less important when CAMHS In-Reach are embedded further into the education system as they may have a deeper understanding of what specific problems services target. Currently though, schools reported selecting providers through their reputations and how well established they were.

Secondly, for services that schools do offer in-house or signpost to, there is still a large demand and long waitlists.

*Young people’s expectations of how long they should have to wait to access support does not match up with the waiting times… young people and adults alike felt that they should be able to access support within a month, even in more moderate cases that doesn’t meet the CAMHS threshold. (*Welsh Youth Parliament, 2020*, p. 12)*

This limits the opportunity to ask students about their service preference, and school staff, health and local authority professionals talked about recurrent service users, who engage with a service, but it does not help improve their problems, so they return for further help. Within education, this was often because school staff were directed by wider education sector staff that adolescents would need to try several lower-level services before being offered further support. This had the unintended consequence of exacerbating waiting lists as young people are encouraged to try more services.

Thirdly, the service offer was dynamic and variable as commissioning processes for voluntary sector organisations/local authority services provided short term funding, and commissioners were always looking for the next approach without evaluating existing provision. Concern was expressed because of the lack of evidence-based interventions (as directed by the WSA). A movement towards funding research about school mental health provision and assessing the evidence base of pervasive programmes delivered in Wales was found, but this was overdue:

*I am absolutely in favour of kind of evidence-based interventions, especially because it is essentially public money going into interventions, which should have a reputable evidence base behind it… But, my god, we've talked about that for years. (Policy and Practice Stakeholder)*

### Addressing the Needs of the “Missing Middle”

Our third research question considered how the “missing middle’s” needs could be addressed by a school-based intervention. This section outlines the potential targets of evidence-based indicated interventions or services for the “missing middle” that were discussed, and how they could be delivered.

#### Potential Targets for Intervention

Potential intervention targets were identified at the intrapersonal and interpersonal levels. At an intrapersonal level, there was a consensus of the need to build resilience and coping mechanisms through improved emotional regulation and problem-solving. In terms of emotional regulation, situations were described where problematic longer-term emotional regulation strategies such as rumination and avoidance resulted in the “missing middle” not participating in social clubs or not attending school. Students felt these strategies were developed as they had many small but cumulative negative experiences or one traumatic event that changed how they emotionally processed future events and activities:

*I think for most people, it could be like a lifechanging situation. So it could be a circumstance where you like were doing fine with your lifestyle and you were going to school and stuff, and then you could have a situation occur that you didn't really think was possible…I feel like someone could spiral really easily because you don't really like prepare you for those sort of events when they do happen. And so that's why it can cause you to become more stressed and like be anxious. (Student)*

School staff agreed and expressed that while training was offered for them to understand safeguarding, self-harm, bereavement and other major life events, they were still not equipped to support the “missing middle” to deal with the residual emotions or anxiety and depression symptoms that led to young people’s distress and impaired functioning:

*I think when we've got a young person displaying those emotions and feelings, we will put in an intervention, mainly myself, and then we'll look for alternatives. So it could be like we've got school-based counsellors. And I know that they might just plug the gap, and I think that's the problem. We plug the gap, but we're not actually getting to the root cause of what's happening. (School Staff)*

In terms of problem-solving as an intervention approach, students thought some young people may not be able to locate why they feel certain emotions (especially if they had not had a major life event) or understand what they need help with, so supporting them to identify their problems and feelings would be a good first step. They also thought problem-solving could reduce waiting lists as young people would know how to solve their own problems. School staff particularly thought equipping students with the skills to approach their own problems and decide on a solution could prevent issues reaching a crisis point.

When discussing how a school-based intervention could address the “missing middle’s” needs (research question 3), some adults questioned locating the causes of depression and anxiety as the emotional regulation and problem-solving skills of adolescents rather than targeting the wider impacts of family disfunction or trauma. Other adults thought where adolescents cannot change a problem themselves, learning how to change their thinking about a problem would help them take back control. Further, students and school staff were concerned that a problem-solving intervention would quickly become overwhelmed as many students would want to access it.

At an interpersonal level, suggested targets of intervention included focusing on reducing loneliness and supporting adolescents to develop healthy social relationships with their peers. As shown above, there was concern that COVID had disrupted young people’s opportunity, and their ability to, develop strong relationships with their peer group. It was felt some young people spent large amounts of their time at home, either alone or on devices, and this social isolation was exacerbated by families that actively tried to keep them at home.

*Navigating what healthy friendships are and that self-worth, "I'm worthy to have good friendships around me, for them to treat me appropriately." And connecting with other- you know, like the friendships, and then from that, you'd be absolutely shocked to hear how many young people just simply don't have any friends. (Policy and Practice Stakeholder)*

Participants advised how they were already informally combating student loneliness, for example, students and staff at one school talked about an initiative they had set up at lunch time for students who did not have friends to come together.

Three wider factors were raised as important when considering intervention for the “missing middle". First, participants said there are links between the interpersonal issues, and emotional regulation and problem-solving as potential tools to overcome these, as students would learn transferable skills about how to rethink the way they emotionally processed a relational problem or reframe how they approach a problem such as loneliness. Second, there is a need to consider whether any targets are suitable for those who are neurodivergent, as it was perceived most interventions were neuronormative. Third, school interventions should be part of a spectrum of support including family services because:

*You know, families, communities have a responsibility as well to do that. Yeah, it's difficult 'cause I know not all families feel resourced to do that. But I think we do need to be supporting families with doing some of this as well. (Policy and Practice Stakeholders)*

#### Potential Delivery

Related to research question 3 about how a school-based intervention could address the needs of the “missing middle”, there was consideration of the delivery agents and format. A recurring talking point from all stakeholders was that all adolescents, but especially those from the “missing middle”, needed a trusted adult within the school setting to support them and be a point of contact when they felt overwhelmed or unhappy. Delivering an intervention within the school setting was popular to accommodate this but consideration of how this was done with those not frequently attending school would be needed. A benefit to school provision was that it would limit the time that adolescents missed from lessons.

This need for a trusted adult also meant that most thought that this intervention should be delivered on a one-to-one basis so adolescents could freely discuss the emotions and problems they were dealing with, and it would need to be confidential:

*P3: And a lot of young people don't want to do group work, do they.*

*P2: If they're isolated, they're anxious.*

*P4: Especially in school if there's other people they know. (Policy and Practice Stakeholders)*

However, there was a consideration that individual work could step up to group work, especially when trying to combat loneliness. Participants worried one-to-one provision would not be feasible due to cost, and high demand would result in long waitlists like other school provision. Demand may particularly be an issue as students wanted the intervention to be available for all year groups as they felt school support was often focused on older students.

A further recommendation in Wales has been that:

*If feasible, external providers should be invited into schools to raise awareness of this [referral processes] with staff and pupils and also to explore potential options for on-site delivery of services. (*Brown et al., 2021*, p. 104)*

Most students and staff agreed that delivery by an external workforce was preferential. This was because staff did not have the time to deliver targeted interventions, even if they were given training, and as most students preferred to have an independent delivery person due to confidentiality, as long as:

*before you actually talk to them about your feelings, you should get, like we've done, we've done icebreaker. We should try and get to know them more because some people might have struggle(s) trusting. (Student)*

The idea of task sharing was discussed with participants as the research team knew delivery by clinical specialists may not be possible. A few delivery workforces were suggested if adequate training and supervision was provided, including extending the remit of CAMHS In-Reach, youth workers, or commissioning voluntary organisations who already deliver non-clinical support. Participants had mixed views on the teaching assistant workforce delivering such an intervention. While they often already deliver this level of provision, it was recognised that remuneration for this workforce was highly variable and not sufficient for the level of responsibility in delivering an intervention to the “missing middle”.

## Discussion

This study explored the Welsh adolescent mental health context and the needs of secondary school students aged 11–18 termed the “missing middle”. It makes a novel contribution to the existing research through addressing Beket et al’s (2025) review conclusion that there is an urgent need to understand how stakeholders’ perceive subthreshold symptoms and the ways that this adolescent population can be supported. It is also an example of the growing literature on university-community partnerships that attempt to involve stakeholder understandings in the development or adaptation of school mental health interventions/services (Lawson & Owens, 2024).

For research question 1, it was found that the “missing middle” are seen as a growing population of adolescents who struggle to access targeted support due to the systematic issues outlined. Certain groups, such as the more economically disadvantaged or care-experienced, were more likely to be within this population, and presentations varied widely from those exhibiting behavioural problems to those that are introverted and hidden. Consensus building research with adolescents, parents, clinicians, service providers and commissioners and policymakers in Australia (Menssink et al., 2025) aligns with our findings in two main ways. First, that the “missing middle” term is not well liked as it focuses on missing adolescents rather than systemic issues due to missing or inaccessible services. Second, certain groups are at higher risk as they are socially excluded.

Variations between these studies were also found including a focus in Australia on the health system plugging the missing service gap, whereas in Wales there has been a focus on bringing services into education settings to provide support for the “missing middle”. Also, other socially excluded groups were thought to be at increased risk, such as Culturally and Linguistically Diverse (equivalent to black and minority ethnic communities in the UK) who were not discussed in the Welsh study. The latter emphasises that both studies are exploratory and rely mainly on stakeholders’ perceptions. Epidemiological research is needed to further understand the “missing middle” including evidence of which individuals or groups are at higher risk to be part of this population, and higher risk to continue onto having diagnosable conditions, so intervention can be prioritised.

It was also found that the premise that the “missing middle” are synonymous with adolescents who present with elevated anxiety and depression symptoms was only partially supported by the data. Findings also showed that the “missing middle” could have high levels of distress or functional impairment without elevated symptomology, or alternatively that there were adolescents who were functioning well educationally but were suffering silently. Other studies have reported an imperfect overlap between impairment and anxiety and depression symptoms with about 1 in 10 adolescents reporting low symptoms but high impairment (Wille et al., 2008). The need to assess functional outcomes in universal adolescent mental health interventions is highlighted elsewhere (Andrews & Schweizer, 2023). The same is true for outcomes in indicated interventions; however, this study implies there is a need to assess functionality during identification/screening processes to include all adolescents that need intervention. There is some evidence from this study that assessment for targeted intervention in Wales does focus on contextualising cases and understanding functioning, but not in a systematic way.

Currently there is a lack of clarity internationally on whether existing indicated interventions use functional screening or assess functional outcomes, their delivery processes, and the extent to which they are neuronormative or include family components. A number of reviews have synthesised the evidence base for adolescent mental health interventions (Caldwell et al., 2019; Clarke et al., 2021; Hetrick et al., 2016; Ssegonja et al., 2019; Werner-Seidler et al., 2021; WHO & UNICEF, 2021) although they often include interventions across the universal, targeted (indicated and selective) and treatment domains, making it difficult to draw out the make-up of indicated interventions. Reviews are also limited by their lack of synthesis on programme theory, and context and implementation data which are key to understanding how an intervention works (Skivington et al., 2021). A follow-up scoping review of indicated school-based interventions (Reed & Shenderovich, 2024) will be conducted to provide clarity on the issues raised, where data exists. This will support the research team to adapt an intervention to Wales that can screen for elevated levels of symptoms or functioning impairment to meet the needs in Wales.

Findings for research question 3 further illustrate the potential targets of and delivery considerations for interventions adapted for the “missing middle” in Wales. Potential targets for intervention focused on the intrapersonal domains of cultivating emotional regulation (improved management of emotions) (Wellcome Trust, 2021) and problem-solving (changing an individual's beliefs, expectations from a negative to more positive stance, and enhancing skills to resolve problems effectively) (Michelson et al., 2022), and the interpersonal levels of reducing loneliness and supporting the development of social relationships. Although caution was expressed about ensuring interventions are not neuronormative and are supplemented by family approaches where problems may be a joint endeavour to change.

In terms of delivery, there was an overall preference to utilise an existing non-clinical workforce, however whether delivery should be at the individual or group level was debated. Group-based indicated programmes have been shown to be effective in reducing symptoms (e.g., Stice et al., 2008) and can be pragmatic for cost and scalability reasons. Adolescents in this study did prefer individual delivery though, and in treatment services the therapeutic alliance or one-on-one relationship with adolescents is an important predictor of outcomes (Campbell & Simmonds, 2011; Labouliere et al., 2017). Balancing intervention acceptability and feasibility considerations will be the focus of later stages of this research.

For research question 2, the study has contributed to understanding the contextual shift to prioritise adolescent mental health in Wales, while highlighting continuing systemic issues. Data showed that adolescent mental health had been prioritised by meeting Shiffman and Smith’s (2007) three conditions for gaining political priority for health issues. These are: political leaders express support through legislation and policy documents (Welsh Government, 2015, 2019, 2021); there is an emphasis on enacting policies by making them statutory requirements, such as the WSA (Welsh Government, 2021); and resources have been allocated to increase funding to provide services for the “missing middle”. Issues remain in terms of whether sufficient funding to the disease burden is available, whether separate assessment structures for community and education sectors maintain siloed work, and how the multiple targeted services that exist should provide clearer theoretical information, be better coordinated, offered equitably, and be evaluated or supplemented by evidence-based programmes from outside Wales as this study intends.

A further challenge in Wales, and more generally, is that this population have varied symptoms and functioning profiles as they are at the intersection of the different but related constructs of mental health and mental wellbeing. The former links to the medical model of health with socially constructed thresholds for diagnosing whether a disorder is present or not (Antonovsky, 1996) and the latter to a social model of mental wellbeing where happiness, purpose in life and functioning are key (Ryan & Deci, 2001). This study highlighted some difficulties with this. For instance, defining this population, which was a main aim of this study as directed by the ADAPT guidance (Moore et al., 2021), was not easy as it is a heterogenic population. As stated above, further epidemiological evidence is needed to understand this population. Secondly, documents and participants often flowed between the mental health and wellbeing paradigms making it difficult sometimes to understand what pertained to all adolescents and what was specific to the “missing middle” e.g., whether it was for universal or targeted prevention.

Lastly, caution is needed not to dismiss young people’s concerns when they seek specialist treatment, but their needs are considered within the realms of prevention or mental wellbeing. This is reflected in this study by the perceived mix of advantageous and detrimental effects of the increased awareness of mental health in Wales. It was believed that increased reporting and help seeking of ‘genuine’ cases that would otherwise be unidentified was accompanied by unintended consequences such as self-diagnosis, as transient low or anxious moods or mild distress were considered diagnosable disorders. This aligns to the dual implications outlined in the prevalence inflation hypothesis (Foulkes & Andrews, 2023). However, this makes having appropriate preventive services available more important, so adolescents receive timely support dependent on their level of need, and rejection from help seeking does not exacerbate their mental health concerns.

## Strengths and Limitations

This study represents the first attempt known to the authors to understand the “missing middle” in Wales. It has included the perspectives of adolescents, school staff, policy, and education, health and voluntary sector practice stakeholders. However, it is a small-scale qualitative study that used purposive and snowball sampling to identify participants, so a key limitation is that other stakeholders may have different perspectives than the sample obtained. Primary data was gathered from stakeholders at three schools with an interest in participating in the research. These schools may have offered more services than is typical. Further, there was a tendency for participants to discuss adolescents with anxiety rather than depression symptoms which is possibly due to the visibility of anxiety over depression symptoms, but for the students involved, could be a consequence of using an anxiety focused vignette.

## Conclusion

While there is a growing population of adolescents with subclinical but elevated anxiety and depression symptoms that indicated programmes target, populations at risk of future disorder onset may also include those with high distress or functional impairment without elevated symptomology. Screening processes linked to functional assessments are key to ensuring all individuals that need intervention are identified. Furthermore, for school-based interventions, adolescents prioritise confidentiality and developing trusting relationships with delivery agents so one-to-one, external provider delivery is preferred, albeit these parameters may be inconsistent with pragmatic considerations such as cost and scalability. Further research is needed to synthesise the existing processes and theoretical make-up of indicated interventions to understand if such issues are attended to in the global indicated intervention field.

## Supplementary Information

Below is the link to the electronic supplementary material.

Supplementary Material 1
