# Supplementary material for: Understanding the Context and Needs of Adolescents Experiencing Subclinical Anxiety and Depression Symptoms in Wales: Document Analysis and Qualitative Data Collection
Source: School Ment Health. 2026 Feb 21;18(2):617–33. doi: 10.1007/s12310-026-09849-3 (PMC13279597; doi:10.1007/s12310-026-09849-3)
Supplement: Supplementary file 1 — Supplementary Material 1 [file 12310_2026_9849_MOESM1_ESM.docx]

**Appendix 1**

**Table 2**

*Document analysis references and types*

| No. | Document Reference and URL | Document Type |
| --- | --- | --- |
| 1 | Brown, R., Van Godwin, J., Edwards, A., Burdon, M., & Moore, G. (2021). *Development of a theory of change and evaluability assessment for the whole school approach to mental health andemotional wellbeing*. <https://www.gov.wales/sites/default/files/statistics-and-research/2021-12/development-of-a-theory-of-change-and-evaluability-assessment-for-the-whole-school-approach-to-mental-health-and-emotional-wellbeing.pdf> | Primary Research |
| 2 | Children, Young People and Education Committee. (2018). *Mind over matter: A report on the stop change needed in emotional and mental health support for children and young people in Wales*. National Assembly for Wales. <https://senedd.wales/media/jr4oyh4p/cr-ld11522-e.pdf> | Report |
| 3 | Children’s Commissioner for Wales. (2020). *Coronavirus and Me*. <https://www.childcomwales.org.uk/wp-content/uploads/2020/06/FINAL_formattedCVRep_EN.pdf> | Report |
| 4 | Children’s Commissioner for Wales. (2020). *No wrong door: Bringing services together to meet children’s needs*. <https://www.childcomwales.org.uk/wp-content/uploads/2020/06/NoWrongDoor_FINAL_EN230620.pdf> | Report |
| 5 | Children’s Commissioner for Wales. (2022). *Making Wales a No Wrong Door Nation - how are we doing?* <https://www.childcomwales.org.uk/wp-content/uploads/2022/02/No-Wrong-Door-Report-February-2022.pdf> | Report |
| 6 | Hewitt, G., Copeland, L., Page, N., Willis, S., Murphy, S, Edwards, A., Jones, S., Renold, R. & Evans, R. (2022). *Review of statutory school and community-based counselling services: Optimisation of services for children and young people aged 11 to 18 years and extension to younger primary school aged children*. Welsh Government.  <https://www.gov.wales/sites/default/files/statistics-and-research/2022-04/review-of-statutory-school-and-community-based-counselling-services-technical-report_0.pdf> | Primary Research |
| 7 | Holtom, D., Lloyd-Jones, S., & Bowen, R. (2021). *Evaluation of the Child and Adolescent Mental Health Service (CAMHS) In-Reach to Schools Pilot Programme: Final Report*. Welsh Government. <https://gov.wales/sites/default/files/statistics-and-research/2021-06/evaluation-of-the-child-and-adolescent-mental-health-service-camhs-in-reach-to-schools-pilot-programme-final-report.pdf> | Primary Research |
| 8 | NHS Executive (2022) *Together for Children and Young People: Legacy Report.* [performanceandimprovement.nhs.wales/functions/strategic-programme-for-mental-health/together-for-children-and-young-people-2/t4cyp-docs/together-for-children-and-young-people-programme-legacy-report/](https://performanceandimprovement.nhs.wales/functions/strategic-programme-for-mental-health/together-for-children-and-young-people-2/t4cyp-docs/together-for-children-and-young-people-programme-legacy-report/) | Report |
| 9 | Powys Teaching Health Board (2019) *Mind Over Matter Report Follow-up: Together for Children and Young People Programme Update.* <https://business.senedd.wales/documents/s87903/CYPE5-15-19%20-%20Paper%20to%20note%203.pdf> | Report |
| 10 | Welsh Government (2020) *The emotional and mental health of children and young people in Wales – next steps for Mind over Matter.* <http://seneddtest.assemblywales.org/documents/s99577/CYPE5-09-20%20-%20Paper%20to%20note%201.pdf> | Report |
| 11 | Welsh Government (2020) *Together for Mental Health: Delivery Plan 2019-2022.* <https://www.gov.wales/sites/default/files/publications/2020-01/together-for-mental-health-delivery-plan-2019-to-2022.pdf> | Policy |
| 12 | *Welsh Government (2021). Framework on embedding a whole-school approach to emotional and mental wellbeing: Guidance* (Guidance Number 269/2021). [*https://gov.wales/framework-embedding-whole-school-approach-emotional-and-mental-wellbeing*](https://gov.wales/framework-embedding-whole-school-approach-emotional-and-mental-wellbeing) | Policy |
| 13 | Welsh Parliament. (2020). *Mind over matter: Two years on*. Children Young People and Education Committee. <https://senedd.wales/laid%20documents/cr-ld13568/cr-ld13568-e.pdf> | Report |
| 14 | Welsh Parliament (2020, December 11). Mental health, young people and the pandemic. *Senedd Research* [Mental health, young pehttps://research.senedd.wales/research-articles/mental-health-young-people-and-the-pandemic/ople and the pandemic](https://research.senedd.wales/research-articles/mental-health-young-people-and-the-pandemic/) | Research Overview |
| 15 | Welsh Parliament Children and Young People is there a mental health crisis? *Senedd Research* <https://research.senedd.wales/research-articles/children-and-young-people-is-there-a-mental-health-crisis/> | Research Overview |
| 16 | Welsh Youth Parliament. (2020). Let’s Talk about Mental Health.  <https://youthparliament.senedd.wales/media/h4wdvpzl/emhs-report-eng.pdf> | Report |

**Appendix 2**

Project Focus Group Schedule for Students

1. Welcome and Introduction

Thank you for joining me here today and agreeing to take part in this focus group. My name is [lead researcher name], and this is my colleague [researcher name], we are researchers from Cardiff University.

We are here today to learn more about what young people understand and think about the needs of young people who may be experiencing increased symptoms of anxiety and depression but so far, have not been diagnosed with a mental health condition. From now on we will refer to these young people as those ‘who are starting to feel a bit unhappy or anxious’.

**During the focus group we will not be asking you to reveal or discuss your own mental health status.** Instead, we will be doing a few activities to help the group talk about:

- The characteristics and needs of young people who may be starting to feel unhappy or anxious.
- What help is available to these young people and whether there are any gaps in help.
- What are the positives and negatives of some programmes the research team have found.

1. Recording and Consent

Even though you have completed a **consent** form, this does not mean you have to take part, and you can change your mind. This focus group will last about 45 minutes, and you can leave at any time, without reason. [name of teacher] is close by, in case anybody wants to leave.

I will be **recording the focus group** so that I can remember everything you talk about, but we’ll only record your voices. This will be kept private, unless you tell me that someone is being hurt and then I will need to pass this information onto your school. However, **we ask you not to** repeat what has been said by other students to anyone outside of this room.

In the focus group I want you to do the talking as I am very interested in hearing what you have to say. We would like **everyone to have the chance to talk** but you do not have to discuss any questions that you do not want to. **There are no right or wrong answers**: Everything you think is really important. I want to hear what everyone has to say.

After the focus group, our conversation will be typed up, but it will be anonymised by removing any information that can identify you.

**Do you have any questions before we get started with the focus group?**

**Start audio-recording.**

1. Ice breaker

So, we will begin by learning a bit more about each other, I’ll start. Perhaps we can start with our names and what we are currently enjoying doing outside of school, a favourite movie/series or hobby? My name is [lead researcher name] and I (…). (Introduce any other colleagues).

1. The characteristics and needs of young people who may be starting to feel a bit unhappy or anxious.

**We first want to introduce you to a scenario of a young person.**

[Scenario to read]: Sam is a 15-year-old who attends your school. They live at home with their mum and dad and their sibling who is 3 years older than them. They have a close group of three friends who they went to primary school with and who are now in the same secondary school. They feel worried about a lot of things including how they look, their schoolwork and their upcoming exams, and what they will do in the future. However, recently they have started to worry about more everyday things and feel like they do not want to leave the house much or spend time with their friends. Sometimes they worry so much that they can feel their heart beating in their chest and they feel a little lightheaded. Their parents have said that all young people feel worried at their age, including their sibling, but now they are fine. This has made them feel unsupported at home, so they don’t want to raise this with their parents again.

1. What do you think that Sam should do?
2. What help do you think that Sam needs?
3. Who do you think Sam should seek help from?

Thinking more generally about young people who are starting to feel a bit unhappy or anxious.

1. Can you tell me a little about who you think these young people are (do they have any similar characteristics/or are there some young people who you think are more likely to start to feel unhappy or anxious?).
2. How many young people in secondary schools do you think are starting to feel unhappy or anxious?
3. What help is available to these young people and whether there are any gaps in help.

**Next, we would like you to tell us about the services available for young people like Sam who are starting to feel a bit unhappy or anxious.**

[Show the students the printout of the socio-ecological model and ask them to write on yellow post-its what support services they know of. Go through the post-its and ask about the services they have listed (if there are many, focus on those linked to school and CAMHS)]:

- 1. Would you suggest that young people use this service – why/why not?
  2. What helps young people to access/use this service? [What stops young people from accessing/using this service?]
  3. Do you think students are happy to seek help within school? (if so, from who- if not, why?).
  4. Do you think the services the school has to offer (i.e., school counsellors) meets the needs of these young people? (if so, how? If not, why?)
  5. Do you think there are any gaps in the services for young people starting to feel unwell? (If so, what?)
  6. If a young person is starting to feel unwell, what support do you wish would be available to them? [How should this look?]

1. What are the positives and negatives of some programmes

**Lastly, we would like you to feedback on some ideas we have to help young people starting to feel unwell. We would like you to tell us what is good and bad about our ideas and we will write them on the flipchart to discuss them.**

- One idea is to teach groups of students about mental health. What do you think of this?

(Follow up questions: who should deliver these groups; do you think young people would want to do this?)

- A second idea would be to run problem-solving counselling with young people who need it. This would entail meeting counsellors so they could help the student to identify the problem, generate and assess options to solve it, and then put one solution into practice, before the student and counsellor review the outcome. This is hoped to build the students skills to do problem-solving on their own in the future. What do you think of this?

(Follow up questions could be: Should this be one-to-one or could it be group based; Should teachers be taught to be a problem-solving counsellor; how about a trained mental health professional recruiting, or training anyone who applied to do this as a job; can you think of anyone else who could do this?)

- Before we finish we wanted to ask one more time – do you have any other ideas of ways we could help this group of young people.

**End audio-recording**

- Close

Thank you for taking part in the focus group, before you leave, we have provided some details of staff in your school or outside organisations you can contact if you want to chat to someone. [Team to remember there is a staff member close by to call on if the young people want to chat to anyone].

Thank you for your time today, we really appreciate it.
